# Application of Indocyanine Green During Arteriovenous Malformation Surgery: Evidence, Techniques, and Practical Pearls

**DOI:** 10.3389/fsurg.2019.00070

**Published:** 2019-12-11

**Authors:** Chase H. Foster, Peter J. Morone, Samuel B. Tomlinson, Aaron A. Cohen-Gadol

**Affiliations:** ^1^Department of Neurological Surgery, George Washington University Hospital, Washington, DC, United States; ^2^Department of Neurological Surgery, Vanderbilt University Medical Center, Vanderbilt University, Nashville, TN, United States; ^3^School of Medicine and Dentistry, University of Rochester Medical Center, Rochester, NY, United States; ^4^Goodman Campbell Brain and Spine, Department of Neurological Surgery, Indiana University, Indianapolis, IN, United States

**Keywords:** AVM, fluorescence, indocyanine green, neurosurgery, resection, videoangiography

## Abstract

Indocyanine green (ICG) is a fluorescent molecule that enables visualization of hemodynamic flow through blood vessels. The first description of its application to the resection of arteriovenous malformations (AVMs) did not occur until 2007. Since then, industry leaders have rapidly integrated this optical technology into the intraoperative microscope, and the use of ICG videoangiography (VA) has since become routine in AVM surgery among some academic centers. A number of case series have been published since the introduction of ICG VA to AVM neurosurgery. These early reports with small sample sizes were largely qualitative, assigning to the technology “usefulness” and “benefit” scores as perceived by the operators. This lack of objectivity prompted the development of FLOW 800 software, a proprietary technology of Carl Zeiss Meditec AG (Oberkochen, Germany) that can quantify relative fluorescence intensity under the microscope to generate color maps and intensity curves for *ad hoc* and *post hoc* analyses, respectively. However, subsequent case series have done little to quantify the effect of ICG VA on outcomes. The available literature predominately concludes that ICG VA, although intuitive to deploy and interpret, is limited by its dependence on direct illumination and visualization. The subcortical components of AVMs represent a natural challenge to ICG-based flow analysis, and the scope of ICG VA has therefore been limited to AVMs with a high proportion of superficial angioarchitecture. As a result, digital subtraction angiography has remained the gold standard for confirming AVM obliteration. In this review, we provide an overview of the existing literature on ICG VA in AVM resection surgery. In addition, we describe our own experiences with ICG VA and AVMs and offer the senior author's surgical pearls for optimizing the marriage of fluorescence flow technology and AVM resection surgery.

## Introduction

A tenant of any neurosurgical operation is the maintenance of perfusion by way of avoiding undue vascular compromise. In the resection of intracerebral arteriovenous malformations (AVMs), this means the exclusion of en passage (i.e., uninvolved) vessels as well as the protection of nidal draining veins until arterial feeders have been disconnected (1). Indocyanine green (ICG) is a non-specific fluorescein-like dye that can be injected systemically to help delineate the margins of vascular anatomy under direct visualization and represents one such maneuver to successfully accomplish these goals. ICG videoangiography (VA) specifically is useful in this endeavor in that it enables the operator to directly and immediately assess the integrity of vessels under the intraoperative microscope.

Since its introduction to the field, the utility of ICG VA in optimizing the margins of safety during AVM resection has become more widely accepted. Now, some authors would posit that ICG VA is a standard component of AVM resection surgery (2). Both single-institution (3) and expert-driven (1) reports of the practicality of ICG VA as an intraoperative armament have been bolstered by larger reviews of the topic by various research cooperatives in recent years (2, 4, 5). A contemporaneous review of the topic is warranted to refresh neurosurgeons' understanding of ICG's important role in neurosurgery as it relates to AVM resection surgery and to provide an updated synthesis of available data.

## History of ICG

ICG dye was first introduced to the field of medicine after its approval by the Food and Drug Administration in 1956 (6). Providers soon discovered it to be helpful in a variety of specialties, beginning with cardiology and ending, for our purposes, in neurosurgery in 1995, when Hongo et al. (7) applied ICG to the study of cerebral hemodynamics. Their seminal work was followed by that of Raabe et al. (8), who in 2003 used ICG VA during the microsurgical clipping of intracerebral aneurysms. The use of ICG VA in neurovascular surgery has become ubiquitous, if not expected, since the subsequent introduction of an ICG VA integrated microscope to the neurosurgical operating room by Carl Zeiss Meditec. Its usefulness for excluding residual nidus in AVM resection has been endorsed repeatedly since the formative work by Takagi et al. (9) in 2007 and by Killory et al. (10) 2 years later. Part of ICG VA's appeal stems from data suggesting that it can produce results somewhat comparable to those of digital subtraction angiography (DSA) (8), which is considered the gold-standard method of assessing AVM resection completeness (11). The relatively recent development of FLOW 800 quantitative software (Carl Zeiss Meditec) has perhaps helped to mitigate some of the often-cited shortcomings of ICG VA (discussed below) by providing a measure of objectivity to this otherwise subjective auxiliary (12).

## ICG Pharmacology

ICG (C_43_H_47_N_2_NaO_6_S_2_) is a water-soluble molecule with a molecular weight of 744 g/mol (13). ICG is similar to the perhaps more familiar fluorescein in that the fluorescent properties of both are exploited to define vascular borders intraoperatively (13). Specifically, the tricarbocyanine structure of ICG is responsible for its near-infrared fluorescence, having peak excitation and emission wavelengths at 800 and 830 nm, respectively (11, 14). ICG becomes highly bound to plasma proteins such as albumin immediately after systemic intravenous injection, with a 95% rate of binding after just 1–2 s (6, 13). Physically, the molecule is too large to cross the blood–brain barrier and thus collects and concentrates only in intravascular compartments without invading surrounding parenchyma (14). ICG travels uninhibited and unfiltered through intracranial vessels after the standard 2-mL (i.e., 5 mg ICG/1 mL H_2_O) bolus (15–17) is injected into a peripheral vein and appears in as little as 2–3 s under the fluorescent surgical microscope, lasting for a matter of several moments (6, 11). It also harbors favorable pharmacokinetic properties for its purpose of highlighting intraluminal flow. The biological activity of ICG is largely nil inside the body; although it is widely distributed, the liver is the only organ to actively take up ICG (6). Furthermore, the toxicity of ICG is low (2), meaning that several rounds of ICG infusion are generally well-tolerated by the majority of patients, and adverse effects occur only very rarely (17). The most likely adverse effect is allergic cross-reaction in patients with hypersensitivity to iodinated compounds; this, too, has been only very rarely documented (16). With its low side effect profile and negligible overall biologic activity (and thus, toxicity) successive rounds of ICG infusions are limited mainly by parameters such as time until already-present videoangiographic fluorescence dissipates. Practically, because ICG may accumulate in tissues where the blood-brain barrier has been disrupted, the consequential pooling of fluorescence in these areas may obfuscate nearby anatomy if multiple rounds of ICG VA are performed (14).

This combination of physical and pharmacological characteristics makes ICG an ideal choice for delineating superficial AVM anatomy and excluding intervening brain parenchyma. Although near-infrared fluoroscopy has been shown to “penetrate several centimeters into tissue” (18, 19), the obfuscation of deeper anatomy by dilated cortical vessels limits its utility during AVM resection to only real-time evaluation of superficial vascular topography (20). This limitation will be discussed further.

## Evidence From and Applications During AVM Resection

The 2007 report by Takagi et al. (9) is the often-cited first publicized use of ICG VA in AVM surgery. In their case report, the authors described the then-novel application of ICG to detect residual nidus during the resection of a Spetzler-Martin grade (SMG) III AVM from a 2-year-old child that was otherwise missed by intraoperative angiography. In their astute recognition of the potential that this newly integrated ICG VA technology might hold, this case report laid the foundation as the first documented use of ICG, and underscored its ancillary use as an immediately available intraoperative adjunct to the angiographic gold standard. Since 2007, several groups of seasoned operators have applied ICG VA to cohorts of patients afflicted with an AVM (1, 3, 10, 12, 15, 21–25).

Killory et al. (10) were the next to publish their experience; they reported their experience with ICG VA used in 10 consecutive AVM resections. Building on the anecdotal success of their predecessors, they effectively applied intraoperative fluorescence and found it subjectively useful for the detection of feeding arteries into the AVM nidus. They also detected a small residual nidus in 1 case. However, they were also the first to draw attention to this technique's major limitation. Namely, deeper vessels could not be visualized with ICG VA in 2 of their patients; those vessels were detected only after they resorted to DSA.

One year later, Ferroli et al. (23) published a case report detailing the use of ICG VA during microvascular decompression for trigeminal neuralgia in a 52-year-old woman in whom a micro-AVM was the unexpected etiology. In this article, they described the usefulness of ICG VA's ability to provide data about flow dynamics of visualized vessels (more specifically, to enable the operator to diagnose the malformation on the basis of an arterialized petrosal vein). Although fluorescent videoangiography did not ultimately surmount the technical challenges of completely resecting this lesion, it did provide data to help the surgeon avoid making the potentially morbid or mortal error of incorrectly ligating the vessel. Their case highlighted a major advantage of ICG VA: its availability and intuitiveness in the operating room, even when its use was not predicted a priori.

That same year, more than 500 miles away, neurosurgeons in Germany reported similar results in 15 patients with an AVM (22). The resolution of resultant videospatial output was graded by researchers in this series, who found ICG injection runs to be of “excellent” quality in the large majority of cases. In 2 of these cases, the ICG VA elucidated information that affected the operative plan, but it failed to fully visualize draining vessels in 1 case (reportedly secondary to the field depth and insufficient illumination).

Nearly 9,000 miles from their German colleagues, Khurana et al. (16) assessed the intuitiveness and usefulness of ICG VA with a “benefit” grade in 4 Australian patients undergoing 5 AVM resection surgeries. The perceived benefit grade was described as a numerical scale from 1 to 4 with the following criteria: “1” indicated that the ICG application was “not useful”; “2” indicated that the fluorescence was seen as “useful, but not affecting surgical management”; a “3” meant that ICG VA was both “useful and affected surgical management”; and a benefit grade of “4” indicated that it provided “false reassurance” (16). ICG VA received a benefit score of 3 in 3 of these patients, indicating that the surgeons found the fluorescence both useful for and influential in surgical management. However, these positive data are tempered by the outcome of the fourth patient; a 22-year-old woman who had a ruptured SMG III insular AVM. ICG VA initially falsely reassured the operators, and a repeat elective resection was necessary (benefit score of 4). In fact, of the 46 operations in which ICG VA was used (which included open aneurysm repair, bypass operations, and tumor resections), this case was the only instance of ICG VA directly misleading the researchers.

Perhaps summarizing 2010's international experience with ICG VA in AVM surgeries, Wang et al. (26) concluded, after retrospective analysis of 43 AVM resections, that no one adjunctive intraoperative modality, ICG VA included, could persistently delineate anatomy and lesional boundaries with the fidelity of DSA. Other groups, such as Taddei et al. (27) on the basis of their own success in 9 AVM operations in India, contrarily maintained that ICG VA might subvert DSA in some cases.

## FLOW 800 Quantification

The first article describing the use of FLOW 800 quantitative software was published in 2011. Just 4 years after the first publication on ICG VA and AVM surgery, Faber et al. (1) sought to address the subjectivity shortcoming of the technology. Prior to their group's contribution to the methodology, flow quantification had been attempted in several ways as far back as 1998 (28) via laser Doppler perfusion imaging in murine models (29), and diffuse optical spectroscopy with concurrent magnetic resonance correlation in both murine and porcine subjects (28, 30). These methods, however, were impractical in their complexity, requiring both sophisticated technologies and extensive time for interpretation. These obstacles made these earlier methods impractical in the operating room, in which rapid yet accurate assessment would be required. FLOW 800 software was developed to this end. The software is currently available on the OPMI Pentero and Kinevo 900 microscopes (12). FLOW 800 is an analytical software integrated into the intraoperative fluorescent microscope that transcribes the visual data obtained during ICG-laden vessel perfusion into arbitrary intensity units, which then are mathematically corrected for variance in native physiologic flow and fed through a proprietary algorithm to generate a color-coded tissue perfusion map in real time (31). The output of this process can only be considered “semi-quantitative” at best, as the arbitrary intensity units that inform the outputted data are themselves imprecise, non-real data (32). This calculated map also provides information about ICG dye intensity, time to change in intensity from 10 to 90% (i.e., “rise time”), and transit time, which is then interpreted post hoc and used to guide further surgical maneuvers (17, 32).

In the report by Faber et al. (1), this technology was applied during 2 AVM resections: an SMG I incidental malformation in a 38-year-old woman's left frontal lobe, and an epileptogenic SMG III lesion in the right temporal lobe of a 26-year-old man. It is notable that the authors specifically stated that their report was limited to these 2 cases because the lesions therein had characteristics amenable to trialing the newly developed color-encoded flow technology. Nevertheless, the authors described their inaugural experience favorably. In one respect, they commended the ergonomics of the FLOW 800 application in the operating room, even for novice users. In another, they found that quantitation of flow provided valuable insight to understanding how hemodynamics into and out of the AVM nidus were affected by ligation and resection as the procedure progressed. However, these advantages were not enough to overcome the inability of ICG VA to provide data about complex 3-dimensional structures; it remains ill-equipped to assess along the z-axis of deep-seated AVMs. As stated by Kalyvas and Spetzler (33), because FLOW 800 output is calculated from ICG VA input, it will inevitably suffer from the same lack of penetrance.

After their influential first report, Takagi et al. (15) and Kamp et al. (31) reported in 2012 on their continued experiences with 27 runs of ICG VA with FLOW 800 analysis and DSA in a series of 11 and 2 AVM resections, respectively. The researchers used ICG VA at various time points during their procedures, split roughly equally between uses before dissection, after arterial feeder clippings, and after dissection, as guided by perceived need and experience. When compared to DSA, ICG VA performed comparably, visualizing nidal filling before dissection and reliably excluding residual nidal filling after resection in 100% of cases. However, even in experienced hands, ICG VA fell short of matching the ability of DSA to visualize arterial feeding vessels (33 vs. 100%, respectively) and venous drainage vessels (89 vs. 100%, respectively). Although the authors praised ICG VA's intraoperative facility, they concluded by noting its inferiority to the DSA gold standard, particularly in regard to the detection of feeders. Furthermore, the small cohort of patients was specifically chosen for the superficial drainage patterns of their lesions, which might have further biased their opinion about the usefulness of ICG VA.

Ng et al. (21) further discussed the limitations of ICG in AVM resection in their retrospective study published the following year. Over 3 years, ICG VA with FLOW 800 quantification was performed 49 times in their cohort of 24 patients with AVMs. Their experience in many ways mirrored that of previous publications. Namely, ICG VA was useful primarily in confirming totality of resection by way of the absence of fluorescent flow through the primary draining vein. The authors found ICG VA in the detection of arterial feeders before dissection less useful than others had reported before them. In this cohort, 15% of predissection ICG runs were considered not useful by researchers because of “a thin layer of adherent clot” (21). Furthermore, although the authors endorsed FLOW 800 quantitative analysis for helping to distinguish between entering and exiting vessels on the basis of flow delay and intensity, they also lamented the need for relatively trivial maneuvers (i.e., removal of cottonoids, blood, and cerebrospinal fluid from the operative field) to prevent inaccurate ICG intensity readings during quantification. This study was unique in that it did not use concomitant DSA in validating the results of ICG VA. A study of 25 AVM surgeries by Ye et al. (25) that same year recapitulated these data closely, reporting that quantification and emulation of transit time was more useful than visualization on normal playback mode alone in differentiating pathologic from native vessels *in situ*.

In 2014, the Spetzler group published what is thought to be the largest consecutive case series of ICG VA in AVM surgery to date (3). They retrospectively reviewed data from 130 patients divided nearly equally into ICG and no-ICG groups that were otherwise similar according to patient and baseline lesion characteristics, and their results revealed no statistically significant difference between the extent of AVM resection or improvement in functional outcome as measured by the modified Rankin score at 6 weeks (12.5 vs. 14.9%, respectively) (3). Despite these equivocal data, the authors maintained that the results were at least in part attributable to the poor penetrance of direct light and consequent ICG fluorescence in deeper vasculature. The study encouraged its continued use in surgery to treat superficially seated AVMs.

A 2015 study of 7 patients who underwent ICG VA–mediated AVM resection by Fukuda et al. (34) resulted in the first detailed quantitative assessment of flow delay (measured by time to half-maximum fluorescence intensity) at various stages of the operation. Their data revealed that these calculated parameters are reliable and intuitive indicators of decreased intranidal flow after sequential and successful feeder clipping. The authors recommended FLOW 800 as reasonable inclusion in the cerebrovascular neurosurgeon's AVM armamentarium given its ability to provide data on changing hemodynamics as the resection progresses. However, a commentary on this study by Kalyvas and Spetzler (33) throttled this notion to a degree by reiterating that FLOW 800 will share the same limitations as non-quantitative ICG VA, because its output also relies on what fluorescence can be directly observed on the surface.

## Spinal AVMs

Vascular malformations of the spinal cord are grossly similar to their intracranial counterparts, yet are also distinct entities that require a specialized surgical approach. There are far fewer available data regarding the application of ICG VA techniques to these lesions in the neurosurgical library because, in part, of their overall lower incidence (35). Nevertheless, several recent case series have described institutional-specific techniques for ICG application to spinal AVMs in larger cohorts (35–37). Similar to the relationship between DSA and ICG VA of cerebral vessels, ICG VA of spinal vasculature is an increasingly employed intraoperative alternative to the “gold standard” of selective spinal angiography. ICG VA has been used adjunctively in the operating room even after preoperative selective spinal angiography was performed, particularly when the lesion of interest has multiple feeders or appears to be primarily irrigated via the indispensable Artery of Adamkiewicz (36, 37). The arguments for and against ICG VA in spinal AVMs are likewise remarkably similar, with advocates championing ICG VA's practicality and ease of rapid intraoperative interpretation to help inform safe surgical resection and preservation of uninvolved angioarchitecture (37). The technique for its infusion during surgery is also similar, and follows a regimented paradigm of a 25 mg bolus diluted in 50 mL of sterile normal saline via a peripheral vein (35, 37). The slightly modified semi-quantitative software, IR800 (also a product of Carl Zeiss Meditec), may also be readily employed through equipped intraoperative surgical microscopes to aid in the often-challenging identification of niduses and fistulas.

These published reports, though limited to single institution case series, reflect on several years of operator experience in applying ICA VA to the resection of spinal AVMs. They represent a small but apparently growing consensus on ICG VA's usefulness to these challenging operations. Although far more research is needed to address the current paucity of published data on the matter, these foundational case series are sparking innovation and unique applications of their own. In a recent study by Hamauchi et al. (38), ICG was used in combination with catheter-based intraoperative super-selective spinal angiography and high frame rate videography to help identify abnormal vasculature in a single thoracic intramedullary AVM. The slow-motion replaying of the captured video data was reportedly influential in the surgeons' resection approach, and provided crucial qualitative feedback in real-time to help guide an effective operation. This report on a single patient and lesion obviously warrants further rigorous investigation to prove its merits, but is representative of the ongoing interest in applying ICG VA to help treat challenging vascular malformations of the spinal cord.

## Surgical Pearls

The limitations previously discussed are perhaps unsurprising to the neurosurgical community at large. The predominately deep-to-superficial anatomical arrangement of intracranial vessels inherently limits any modality that relies on direct visualization and illumination. Today, no means to fully rectify this limitation has been found, but it still might be attenuated by a combination of operator experience and deft applications of the technology. For example, in 2017, one such experienced group published a video documenting their surgical treatment of a large, eloquently located frontoparietal SMG III AVM in a 31-year-old man who presented with hemiparesis after the malformation ruptured (24). The challenges of the case included the largely subcortical focus of the AVM and its proximity to the central sulcus. Despite these challenges, the surgeons prudently used ICG VA to detect a superficial arterialized draining vein from the lesion and simultaneously differentiated it from the nearby vein of Trolard. The authors used this finding to navigate a subcortical dissection path for the complete resection of the AVM per standard resection techniques. Aptly named the “safe sulcus” approach, this clever application of ICG VA exploits its aforementioned superficial limitation to identify an appropriate starting point for subcortical dissection. This approach has since been adopted by other groups with similar success (39). It represents a novel application of a no-longer-novel neurosurgical technology.

**Figure 1:**
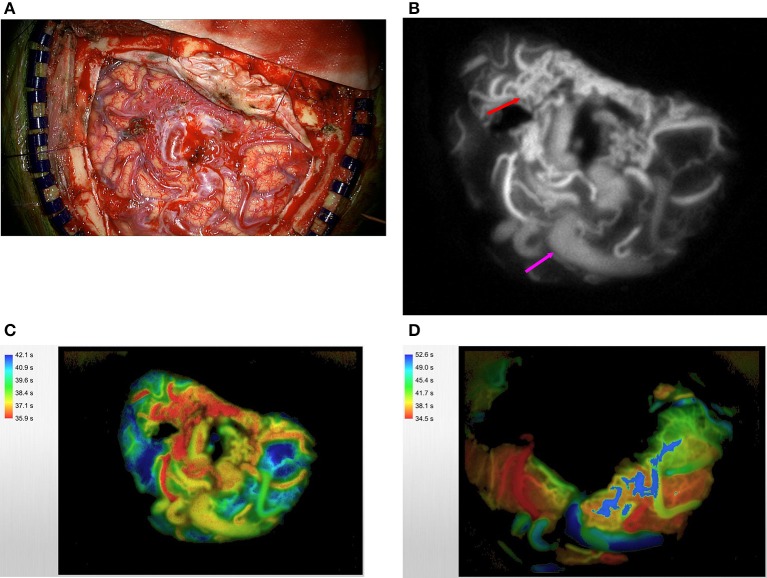
A large right temporoparietal AVM is exposed **(A)**. ICG videoangiography **(B)** demonstrates the early filling of the arterial feeders (red arrow) and delayed filling of arterialized veins (purple arrow); this technique can confirm the identity of these vessels more accurately especially if they are covered by thick arachnoid bands. This information can also guide the early steps of disconnection to start in the area where the concentration of feeding arteries is most (red arrow). The same AVM was analyzed via the use of FLOW 800 software. Please note the feeding arteries in red, arterialized veins in yellow, and normal veins in blue **(C)**. Post-resection FLOW 800 analysis demonstrates that the primary vein is now blue **(D)**.

The senior author recently published his experience using ICG VA in a small group of patients who were undergoing cerebrovascular neurosurgery (12) (Figure 1). FLOW 800 software was used in 10 patients with an AVM in this case series of 23 for the purpose of guiding intraoperative planning and progression. It is the senior author's practice to use only the color maps that are output after semi-quantification because the fluorescence curves that are produced, although valuable in their own right, are not amenable to rapid intraoperative evaluation and thereby extend the patient's time in surgery. ICG was injected intravenously before dissection of the malformation to delineate the angioarchitecture of the lesion and to reliably differentiate involved structures including the feeding arteries from arterialized veins and the uninvolved *en passage* vessels. Of note, to accurately evaluate ICG VA intraoperatively, it is necessary to position the operative microscope at a predefined distance from the operative field and adjust the zoom and focus accordingly. These variables are dependent upon the microscope, and one should consult the microscope's user manual prior to the case.

In 2 of these cases, careful study of the resultant maps enabled the disconnection of an arterialized draining vein and preservation of crucial normal venous drainage. In another illustrative case of a medial parasagittal AVM, mobilization of the hemisphere was made possible after ICG VA enabled the operator to discern high-throughput cortical arterialized bridging veins from those less vigorously irrigated and therefore safe for sacrifice. To our knowledge, this application of the technology is not represented elsewhere in the literature. Although it requires a coupling of comfort with the interhemispheric approach and confidence in interpreting color map data, quantified ICG VA can be cautiously adapted for this purpose.

Nuanced quantitative ICG VA color mapping is particularly useful in a subset of AVMs with specific characteristics, as previous studies have concluded. Intraoperative fluorescent flow analysis directly influenced dissection strategies in each of the cases in the senior author's study. We find ICG VA to be most effective when judicious patient and lesion selection has occurred. For example, superficial AVM anatomy may appear obscured to the naked eye because of overlying dense arachnoid bands. In these cases, ICG VA makes an important contribution to the surgical stratagem by providing real-time data on vascular structures that would otherwise have to be dissected blindly and therefore haphazardly, guided by direct visualization alone. It is also regularly used, in our experience, when there is concern for inadvertent clip-induced *en passage* vessel stenosis. The integrated FLOW 800–capable operative microscope makes evaluation in these cases especially easy by obviating the need to rearrange surgical equipment during the operation. In fact, at least 1 study found ICG VA to be statistically significantly faster to deploy than DSA (34).

The additional time needed to perform DSA might nevertheless be warranted by the equipoise of safety in some situations. For example, in a 2013 article, Kono et al. (40) described the intra-arterial infusion of ICG into the common carotid and vertebral arteries. Although this technique successfully differentiated AVM feeding vessels from normal native arteries, we opine that the additional risk of arterial catheterization in the neck likely nullifies the benefit gained and can be performed more safely via standard DSA. For these reasons, we respectfully do not recommend this form of ICG VA technique.

**Figure 2:**
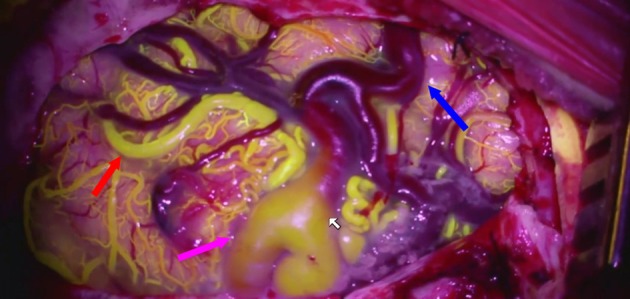
Fluorescein videoangioram of a parietal AVM demonstrates the relative flow in different components of the AVM: feeding artery (red arrow), arterialized vein (purple arrow), and normal vein (blue arrow).

Our team has also used fluorescein in place of the ICG and has found reasonable results similar to those of the ICG (41, 42). The advantage of fluorescein over ICG is that the dye can be visualized through the microscope's binoculars (Figure 2) (43).

Altogether, these data make a strong argument for the role of ICG VA as an adjunct modality in AVM surgery, fulfilling a specific purpose with relative ease in a specific group of lesions. This general gestalt seems to be shared by other experienced operators at high-volume centers (3, 33). Despite the strides made toward objectifying ICG VA with FLOW 800 and the new applications being conceived by shrewd surgeons, current data suggest that ICG VA cannot emulate AVM anatomy with fidelity superior to that of DSA, especially for subcortical structures.

## Conclusion

Studies unanimously agree that ICG VA with FLOW 800 quantitative analysis is an accurate method of delineating superficial AVM structures, but it suffers in regard to deeper structures. In our opinion and experience, it can be used effectively to guide cortical entry points for subcortical dissections and to inform the safe sacrifice of tethering secondary cortical arterialized veins in certain surgical approaches. Likewise, the generated color maps of FLOW 800 are practical, easily interpreted intraoperatively, and sufficient for guiding surgical resection. Although ICG VA has advantages over DSA in terms of deployment speed and ease of use, its inability to visualize subcortical AVM components remains a looming obstacle. Therefore, DSA remains the gold-standard method of confirming complete AVM occlusion (33).

Only 1 systematic review (4) and 2 general reviews (2, 5) of the data on ICG VA in AVM surgery exist in the neurosurgical literature. The systematic review by Scerrati et al. (4), which was published in 2014 and included 7 of the AVM studies available at the time, succinctly concluded that ICG VA might portend a “modest benefit” during AVM resection. Indeed, there is a paucity of adequately powered, prospective, multicenter studies that have investigated the true effect size that this fluorescent technology has on various outcome measures. Although current data on the topic are an amalgam of anecdotes and subjectivity, experienced groups of surgeons worldwide seem to agree that the low toxicity, non-invasiveness, intuitiveness, and real-time feedback offered by ICG VA make it a reasonable tool in the cerebrovascular neurosurgeon's repertoire for open AVM resection. Although the margins and magnitude of this role have yet to be established, it is a meritorious goal for future research.

## Author Contributions

CF performed literature review, synthesized data, and prepared the draft of the final manuscript. PM reviewed, edited, and augmented the drafted manuscript. ST assisted with gathering and contributing data for the drafting of the manuscript. AC-G reviewed and edited the drafted manuscript, provided expertise to technical portions of the manuscript, and provided supervisory support.

### Conflict of Interest

AC-G is a consultant for Carl Zeiss Meditec AG. The remaining authors declare that the research was conducted in the absence of any commercial or financial relationships that could be construed as a potential conflict of interest.

## Abbreviations

AVM: arteriovenous malformation
DSA: digital subtraction angiography
ICG: indocyanine green
SMG: Spetzler-Martin grade
VA: videoangiography.

## References

[1] Faber F Thon N Fesl G Rachinger W Guckler R Tonn JC. Enhanced analysis of intracerebral arterioveneous malformations by the intraoperative use of analytical indocyanine green videoangiography: technical note. Acta Neurochir. (2011) 153:2181–7. 10.1007/s00701-011-1141-z 21894496

[2] Cenzato M Dones F Boeris D Marcati E Fratianni A Crisa FM. Contemporary tools in arteriovenous malformations surgery. J Neurosurg Sci. (2018) 62:467–77. 10.23736/S0390-5616.18.04398-9 29582974

[3] Zaidi HA Abla AA Nakaji P Chowdhry SA Albuquerque FC Spetzler RF. Indocyanine green angiography in the surgical management of cerebral arteriovenous malformations: lessons learned in 130 consecutive cases. Neurosurgery. (2014) 10:246–51. 10.1227/NEU.0000000000000318 24535264

[4] Scerrati A Della Pepa GM Conforti G Sabatino G Puca A Albanese A. Indocyanine green video-angiography in neurosurgery: a glance beyond vascular applications. Clin Neurol Neurosurg. (2014) 124:106–13. 10.1016/j.clineuro.2014.06.032 25033322

[5] Balamurugan S Agrawal A Kato Y Sano H. Intra operative indocyanine green video-angiography in cerebrovascular surgery: An overview with review of literature. Asian J Neurosur. (2011) 6:88–93. 10.4103/1793-5482.92168 22347330 PMC3277076

[6] Bervini D Raabe A Principles of indocyanine green videoangiography. In: Hadjipanayis CG Stummer W, editors. Fluorescence-Guided Neurosurgery: Neuro-oncology and Cerebrovascular Applications. 1st ed New York, NY: Thieme Medical Publishers, Inc. (2018). p. 125–31.

[7] Hongo K Kobayashi S Okudera H Hokama M Nakagawa F. Noninvasive cerebral optical spectroscopy: depth-resolved measurements of cerebral haemodynamics using indocyanine green. Neurol Res. (1995) 17:89–93. 10.1080/01616412.1995.11740293 7609855

[8] Raabe A Beck J Gerlach R Zimmermann M Seifert V. Near-infrared indocyanine green video angiography: a new method for intraoperative assessment of vascular flow. Neurosurgery. (2003) 52:132–9. 10.1227/00006123-200301000-00017 12493110

[9] Takagi Y Kikuta K Nozaki K Sawamura K Hashimoto N. Detection of a residual nidus by surgical microscope-integrated intraoperative near-infrared indocyanine green videoangiography in a child with a cerebral arteriovenous malformation. J Neurosurg. (2007) 107:416–8. 10.3171/PED-07/11/416 18459907

[10] Killory BD Nakaji P Gonzales LF Ponce FA Wait SD Spetzler RF. Prospective evaluation of surgical microscope-integrated intraoperative near-infrared indocyanine green angiography during cerebral arteriovenous malformation surgery. Neurosurgery. (2009) 65:456–62. 10.1227/01.NEU.0000346649.48114.3A 19687689

[11] Yeung JT Kalani MY Nakaji P Applications of indocyanine green video angiography in neurovascular surgery. In: Spetzler RF Kalani MYS Nakaji P, editors. Neurovascular Surgery. 2nd ed. New York, NY: Thieme Medical Publishers, Inc. (2015). p. 194–200.

[12] Shah KJ Cohen-Gadol AA. The application of FLOW 800 ICG videoangiography color maps for neurovascular surgery and intraoperative decision making. World Neurosurg. (2018) 122:e186–97. 10.1016/j.wneu.2018.09.195 30292668

[13] Pogue BW Gibbs-Strauss S Valdes PA Samkoe K Roberts DW Paulsen KD. Review of neurosurgical fluorescence imaging methodologies. IEEE J Sel Top Quantum Electron. (2010) 16:493–505. 10.1109/JSTQE.2009.2034541 20671936 PMC2910912

[14] Roberts DW Valdes PA Indocyanine green. In: Bernstein M Berger MS, editors. Neuro-Oncology: The Essentials. 3rd ed. New York, NY: Thieme Medical Publishers, Inc. (2014). p. 136–46.

[15] Takagi Y Sawamura K Hashimoto N Miyamoto S. Evaluation of serial intraoperative surgical microscope-integrated intraoperative near-infrared indocyanine green videoangiography in patients with cerebral arteriovenous malformations. Neurosurgery. (2012) 70:34–42. 10.1227/NEU.0b013e31822d9749 21768916

[16] Khurana VG Seow K Duke D. Intuitiveness, quality and utility of intraoperative fluorescence videoangiography: Australian neurosurgical experience. Brit J Neurosurg. (2010) 24:163–72. 10.3109/02688690903518247 20121384

[17] Kato N Prinz V Dengler J Vajkoczy P. Blood flow assessment of arteriovenous malformations using intraoperative indocyanine green videoangiography. Stroke Res Treat. (2019) 2019:7292304. 10.1155/2019/7292304 31007890 PMC6441520

[18] Desmettre T Devoisselle JM Mordon S. Fluorescence properties and metabolic features of indocyanine green (ICG) as related to angiography. Surv Ophthalmol. (2000) 45:15–27. 10.1016/S0039-6257(00)00123-5 10946079

[19] Liebert A Wabnitz H Obrig H Erdmann R Moller M Macdonald R. Non-invasive detection of fluorescence from exogenous chromophores in the adult human brain. Neuroimage. (2006) 31:600–8. 10.1016/j.neuroimage.2005.12.046 16478666

[20] Mascitelli JR Burkhardt J-K Lawton MT Indocyanine green videoangiography and arteriovenous malformations. In: Hadjipanayis CG Stummer W, editors. Fluorescence-Guided Neurosurgery: Neuro-Oncology and Cerebrovascular Applications. 1st ed. New York, NY: Thieme Medical Publishers, Inc. (2018). p. 133–40.

[21] Ng YP King NK Wan KR Wang E Ng I. Uses and limitations of indocyanine green videoangiography for flow analysis in arteriovenous malformation surgery. J Clin Neurosci. (2013) 20:224–32. 10.1016/j.jocn.2011.12.038 23277126

[22] Hanggi D Etminan N Steiger HJ. The impact of microscope-integrated intraoperative near-infrared indocyanine green videoangiography on surgery of arteriovenous malformations and dural arteriovenous fistulae. Neurosurgery. (2010) 67:1094–103. 10.1227/NEU.0b013e3181eb5049 20881574

[23] Ferroli P Acerbi F Broggi M Broggi G. Arteriovenous micromalformation of the trigeminal root: intraoperative diagnosis with indocyanine green videoangiography: case report. Neurosurgery. (2010) 67:(3 Suppl Operative):onsE309–10; discussion: onsE10. 10.1227/01.NEU.0000381769.15291.4C 20679916

[24] Rustemi O Scienza R Della Puppa A. Utility of indocyanine green videoangiography in subcortical arteriovenous malformation resection. Neurosurg Focus. (2017) 43:V10. 10.3171/2017.7.FocusVid.1774 28669272

[25] Ye X Liu XJ Ma L Liu LT Wang WL Wang S. Clinical values of intraoperative indocyanine green fluorescence video angiography with Flow 800 software in cerebrovascular surgery. Chin Med J. (2013) 126:4232–7. 10.3760/cma.j.issn.0366-6999.20131649 24238503

[26] Wang S Liu L Zhao YL Zhang D Wang R Zhao JZ. Strategy for assisted cerebral arteriovenous malformation surgery. Zhonghua yi xue za zhi. (2010) 90:869–73. 20646502

[27] Taddei G Tommasi CD Ricci A Galzio RJ. Arteriovenous malformations and intraoperative indocyanine green videoangiography: preliminary experience. Neurology India. (2011) 59:97–100. 10.4103/0028-3886.76878 21339672

[28] Kuebler WM Sckell A Habler O Kleen M Kuhnle GE Welte M. Noninvasive measurement of regional cerebral blood flow by near-infrared spectroscopy and indocyanine green. J Cerebral Blood Flow Metab. (1998) 18:445–56. 10.1097/00004647-199804000-00013 9538910

[29] Wuestenfeld JC Herold J Niese U Kappert U Schmeisser A Strasser RH. Indocyanine green angiography: a new method to quantify collateral flow in mice. J Vascular Surg. (2008) 48:1315–21. 10.1016/j.jvs.2008.06.049 18829217

[30] Cuccia DJ Bevilacqua F Durkin AJ Merritt S Tromberg BJ Gulsen G. *In vivo* quantification of optical contrast agent dynamics in rat tumors by use of diffuse optical spectroscopy with magnetic resonance imaging coregistration. Appl Optics. (2003) 42:2940–50. 10.1364/AO.42.002940 12790443

[31] Kamp MA Slotty P Turowski B Etminan N Steiger HJ Hanggi D. Microscope-integrated quantitative analysis of intraoperative indocyanine green fluorescence angiography for blood flow assessment: first experience in 30 patients. Neurosurgery. (2012) 7065–73. 10.1227/NEU.0b013e31822f7d7c 21811190

[32] Prinz V Hecht N Kato N Vajkoczy P FLOW 800 allows visualization of hemodynamic changes after extracranial-to-intracranial bypass surgery but not assessment of quantitative perfusion or flow. Neurosurgery. (2014) 10(Suppl. 2):231–8; discussion: 8–9. 10.1227/NEU.0000000000000277 24335820

[33] Kalyvas J Spetzler RF. Does FLOW 800 technology improve the utility of indocyanine green videoangiography in cerebral arteriovenous malformation surgery? World Neurosurgery. (2015) 83:147–8. 10.1016/j.wneu.2014.09.010 25219581

[34] Fukuda K Kataoka H Nakajima N Masuoka J Satow T Iihara K. Efficacy of FLOW 800 with indocyanine green videoangiography for the quantitative assessment of flow dynamics in cerebral arteriovenous malformation surgery. World Neurosurgery. (2015) 83:203–10. 10.1016/j.wneu.2014.07.012 25045789

[35] Rangel-Castilla L Russin JJ Zaidi HA Martinez-Del-Campo E Park MS Albuquerque FC. Contemporary management of spinal AVFs and AVMs: lessons learned from 110 cases. Neurosurgical Focus. (2014) 37:E14. 10.3171/2014.7.FOCUS14236 25175433

[36] Walsh DC Zebian B Tolias CM Gullan RW. Intraoperative indocyanine green video-angiography as an aid to the microsurgical treatment of spinal vascular malformations. Brit J Neurosurg. (2014) 28:259–66. 10.3109/02688697.2013.829556 23957775

[37] Jing L Su W Guo Y Sun Z Wang J Wang G. Microsurgical treatment and outcomes of spinal arteriovenous lesions: learned from consecutive series of 105 lesions. J Clin. (2017) 46:141–7. 10.1016/j.jocn.2017.09.003 28986150

[38] Hamauchi S Osanai T Seki T Kawabori M Okamoto M Hida K. Intraoperative real-time identification of abnormal vessels within the bright field by superselective arterial injection of saline and its slow-motion recording using a high frame rate digital camera during surgical treatment of spinal arteriovenous shunts: technical note. J Neurosurg Spine. (2018) 29:576–81. 10.3171/2018.3.SPINE1854 30095384

[39] Muhammad S Niemela M Lehecka M. Utility of video indocyanine angiography to detect the cortical entry point of a draining vein with a superficial vein during arteriovenous malformation surgery. World Neurosurgery. (2019) 122:428. 10.1016/j.wneu.2018.11.054 30448578

[40] Kono K Uka A Mori M Haga S Hamada Y Nagata S. Intra-arterial injection of indocyanine green in cerebral arteriovenous malformation surgery. Turkish Neurosurg. (2013) 23:676–9. 10.5137/1019-5149.JTN.6420-12.0 24101318

[41] Lane BC Cohen-Gadol AA. A prospective study of microscope-integrated intraoperative fluorescein videoangiography during arteriovenous malformation surgery: preliminary results. Neurosurg Focus. (2014) 36:E15. 10.3171/2013.11.FOCUS13483 24484253

[42] Lane BC Cohen-Gadol AA. Fluorescein fluorescence use in the management of intracranial neoplastic and vascular lesions: a review and report of a new technique. Curr Drug Discov Technol. (2013) 10:160–9. 10.2174/1570163811310020009 23363235

[43] Rey-Dios R Cohen-Gadol AA. Technical principles and neurosurgical applications of fluorescein fluorescence using a microscope-integrated fluorescence module. Acta Neurochir. (2013) 155:701–6. 10.1007/s00701-013-1635-y 23392589

